# RELATIONSHIPS OF CEREBRAL PERFUSION WITH GAIT SPEED ACROSS AGE AND SYSTOLIC BLOOD PRESSURE LEVELS

**DOI:** 10.1093/geroni/igac059.2596

**Published:** 2022-12-20

**Authors:** Beverly Gwen Windham, Michael Griswold, Kevin Sullivan, Thomas Mosley, Michelle Mielke, David Knopman, Ron Petersen, Prashanthi Vemuri

**Affiliations:** UMMC-The MIND Center, Jackson, Mississippi, United States; The MIND Center at UMMC, Jackson, Mississippi, United States; UMMC-The MIND Center, Jackson, Mississippi, United States; UMMC-The MIND Center, Jackson, Mississippi, United States; Mayo Clinic, Rochester, Minnesota, United States; Department of Neurology, Mayo Clinic, Rochester, Minnesota, United States; Mayo Clinic Rochester, Rochester, Minnesota, United States; Mayo Clinic Rochester, Rochester, Minnesota, United States

## Abstract

We hypothesized that poorer cerebral perfusion could impair mobility, particularly at lower systolic blood pressure (SBP). Cerebral perfusion was measured via arterial spin-labelled (ASL)-MRI in community-dwelling adults of the Mayo Clinic Study of Aging. Usual-pace gait speed was assessed over 5.6 meters on an electronic mat. Linear regression models estimated cross-sectional gait speed associations with ASL and modifying effects of age and SBP, adjusting for sex and body mass index. Results report differences in gait speed (meter/second) per standard deviation (SD) lower ASL or SBP (mean=139.1 mmHg, SD=19.7 mmHg). Among 479 participants (aged 31-94 years; mean age 67.6 years; 44% women; mean gait speed 1.17m/s), relationships of ASL with gait speed varied by age and SBP (ASL-x-age interaction: p<.007; ASL-x-SBP interaction: p<.007). At age 65, 75 or 85 years and a SBP of 120mmHg, each SD lower ASL was associated with a 0.04 m/s (0.01,0.07), 0.07 m/s (0.04,0.10), and 0.09 m/s (0.05,0.13) slower gait speed at respective ages. At age 65, 75 or 85 years with low ASL (1 SD below the mean), each SD lower SBP was associated with 0.06 (0.02,0.09) m/s, 0.03 (0.01,0.06) m/s or .01 (-0.02,0.04) m/s slower gait speed, respectively. With high ASL (mean ASL+1SD), lower SBP trended towards faster gait at older age, e.g. estimated gait speed was 0.04 m/s (0.00,0.09) faster for each SD lower SBP at age 85years. Poorer cerebral perfusion may contribute to slower gait, particularly with lower SBP. The interrelations of cerebral perfusion, SBP, and age with mobility merit further study.

